# Supplementary material for: Modeling glioblastoma heterogeneity as a dynamic network of cell states
Source: Mol Syst Biol. 2021 Sep 16;17(9):e10105. doi: 10.15252/msb.202010105 (PMC8444284; doi:10.15252/msb.202010105)
Supplement: Supplementary file 5 — Source Data for Figure 3 [file MSB-17-e10105-s001.zip › Figure3A_sourcedata/GSEA_3065/hallmarks_state5.GseaPreranked.1623416302988/HALLMARK_PEROXISOME.html]

Details for gene set HALLMARK\_PEROXISOME[GSEA]

|  || Dataset | state5 |
| Phenotype | NoPhenotypeAvailable |
| Upregulated in class | na\_neg |
| GeneSet | HALLMARK\_PEROXISOME |
| Enrichment Score (ES) | -0.44038668 |
| Normalized Enrichment Score (NES) | -1.4368565 |
| Nominal p-value | 0.028833551 |
| FDR q-value | 0.073447146 |
| FWER p-Value | 0.629 |
Table: GSEA Results Summary

  

Fig 1: Enrichment plot: HALLMARK\_PEROXISOME      
 Profile of the Running ES Score & Positions of GeneSet Members on the Rank Ordered List

  

| PROBE | GENE SYMBOL | GENE\_TITLE | RANK IN GENE LIST | RANK METRIC SCORE | RUNNING ES | CORE ENRICHMENT || 1 | RDH11 |  |  | 224 | 0.422 | 0.0080 | No |
| 2 | CADM1 |  |  | 307 | 0.378 | 0.0286 | No |
| 3 | IDH1 |  |  | 793 | 0.242 | -0.0067 | No |
| 4 | DHCR24 |  |  | 795 | 0.242 | 0.0122 | No |
| 5 | HSD17B11 |  |  | 949 | 0.219 | 0.0122 | No |
| 6 | ATXN1 |  |  | 1062 | 0.203 | 0.0156 | No |
| 7 | FADS1 |  |  | 1148 | 0.191 | 0.0211 | No |
| 8 | CLN6 |  |  | 1301 | 0.171 | 0.0176 | No |
| 9 | ELOVL5 |  |  | 1320 | 0.169 | 0.0288 | No |
| 10 | ABCC5 |  |  | 1456 | 0.157 | 0.0260 | No |
| 11 | LONP2 |  |  | 1460 | 0.156 | 0.0380 | No |
| 12 | MLYCD |  |  | 1590 | 0.142 | 0.0347 | No |
| 13 | SLC35B2 |  |  | 1715 | 0.132 | 0.0312 | No |
| 14 | SLC23A2 |  |  | 1778 | 0.128 | 0.0343 | No |
| 15 | PEX13 |  |  | 1855 | 0.122 | 0.0354 | No |
| 16 | SEMA3C |  |  | 1870 | 0.121 | 0.0433 | No |
| 17 | CLN8 |  |  | 2056 | 0.108 | 0.0311 | No |
| 18 | GSTK1 |  |  | 2283 | 0.093 | 0.0130 | No |
| 19 | GNPAT |  |  | 2476 | 0.081 | -0.0021 | No |
| 20 | ACOX1 |  |  | 2700 | 0.068 | -0.0218 | No |
| 21 | FDPS |  |  | 2849 | 0.060 | -0.0337 | No |
| 22 | CTPS1 |  |  | 3314 | 0.036 | -0.0829 | No |
| 23 | HSD17B4 |  |  | 3981 | 0.008 | -0.1570 | No |
| 24 | PEX6 |  |  | 4191 | -0.001 | -0.1803 | No |
| 25 | IDE |  |  | 4301 | -0.006 | -0.1921 | No |
| 26 | CDK7 |  |  | 4349 | -0.008 | -0.1967 | No |
| 27 | ERCC3 |  |  | 4795 | -0.025 | -0.2446 | No |
| 28 | ACOT8 |  |  | 4827 | -0.027 | -0.2460 | No |
| 29 | ACSL1 |  |  | 4862 | -0.028 | -0.2476 | No |
| 30 | ABCD3 |  |  | 5045 | -0.034 | -0.2653 | No |
| 31 | VPS4B |  |  | 5105 | -0.037 | -0.2690 | No |
| 32 | PEX11B |  |  | 5136 | -0.038 | -0.2694 | No |
| 33 | SLC25A17 |  |  | 5172 | -0.039 | -0.2702 | No |
| 34 | SIAH1 |  |  | 5236 | -0.042 | -0.2740 | No |
| 35 | HMGCL |  |  | 5270 | -0.043 | -0.2743 | No |
| 36 | CAT |  |  | 5358 | -0.047 | -0.2804 | No |
| 37 | ISOC1 |  |  | 5369 | -0.047 | -0.2778 | No |
| 38 | BCL10 |  |  | 5406 | -0.049 | -0.2780 | No |
| 39 | MSH2 |  |  | 5621 | -0.059 | -0.2974 | No |
| 40 | TOP2A |  |  | 5722 | -0.063 | -0.3036 | No |
| 41 | ALDH9A1 |  |  | 6006 | -0.074 | -0.3295 | No |
| 42 | ACSL4 |  |  | 6041 | -0.076 | -0.3273 | No |
| 43 | MVP |  |  | 6150 | -0.082 | -0.3330 | No |
| 44 | SMARCC1 |  |  | 6547 | -0.105 | -0.3691 | No |
| 45 | PEX2 |  |  | 6580 | -0.107 | -0.3643 | No |
| 46 | ACAA1 |  |  | 6891 | -0.128 | -0.3890 | No |
| 47 | CRAT |  |  | 6922 | -0.131 | -0.3821 | No |
| 48 | SOD2 |  |  | 6961 | -0.134 | -0.3758 | No |
| 49 | IDI1 |  |  | 7337 | -0.172 | -0.4043 | No |
| 50 | CTBP1 |  |  | 7648 | -0.211 | -0.4225 | Yes |
| 51 | ERCC1 |  |  | 7735 | -0.223 | -0.4146 | Yes |
| 52 | ECI2 |  |  | 7776 | -0.229 | -0.4010 | Yes |
| 53 | SLC25A4 |  |  | 8128 | -0.303 | -0.4166 | Yes |
| 54 | ECH1 |  |  | 8238 | -0.335 | -0.4025 | Yes |
| 55 | TSPO |  |  | 8443 | -0.404 | -0.3935 | Yes |
| 56 | ITGB1BP1 |  |  | 8459 | -0.413 | -0.3627 | Yes |
| 57 | IDH2 |  |  | 8498 | -0.434 | -0.3329 | Yes |
| 58 | SCP2 |  |  | 8513 | -0.442 | -0.2997 | Yes |
| 59 | CNBP |  |  | 8565 | -0.466 | -0.2688 | Yes |
| 60 | PRDX1 |  |  | 8584 | -0.476 | -0.2333 | Yes |
| 61 | PABPC1 |  |  | 8598 | -0.488 | -0.1965 | Yes |
| 62 | HRAS |  |  | 8633 | -0.514 | -0.1599 | Yes |
| 63 | FIS1 |  |  | 8672 | -0.540 | -0.1217 | Yes |
| 64 | YWHAH |  |  | 8726 | -0.587 | -0.0815 | Yes |
| 65 | PRDX5 |  |  | 8836 | -0.693 | -0.0392 | Yes |
| 66 | SOD1 |  |  | 8848 | -0.709 | 0.0152 | Yes |
Table: GSEA details [plain text format]

  

Fig 2: HALLMARK\_PEROXISOME: Random ES distribution      
 Gene set null distribution of ES for **HALLMARK\_PEROXISOME**
